# A dataset of ambient sensors in a meeting room for activity recognition

**DOI:** 10.1038/s41597-024-03344-7

**Published:** 2024-05-21

**Authors:** Hyunju Kim, Geon Kim, Taehoon Lee, Kisoo Kim, Dongman Lee

**Affiliations:** Korea Advanced Institute of Science and Technology, School of Computing, Daejeon, 34141 South Korea

**Keywords:** Research data, Scientific community, Social sciences

## Abstract

As IoT technology advances, using machine learning to detect user activities emerges as a promising strategy for delivering a variety of smart services. It is essential to have access to high-quality data that also respects privacy concerns and data streams from ambient sensors in the surrounding environment meet this requirement. However, despite growing interest in research, there is a noticeable lack of datasets from ambient sensors designed for public spaces, as opposed to those for private settings. To bridge this gap, we design the DOO-RE dataset within an actual meeting room environment, equipped with three types of ambient sensors: those triggered by actuators, users, and the environment itself. This dataset is compiled from the activities of over twenty students throughout a period of four months. DOO-RE provides reliable and purpose-oriented activity data in a public setting, with activity labels verified by multiple annotators through a process of cross-validation to guarantee data integrity. DOO-RE categorizes nine different types of activities and facilitates the study of both single and group activities. We are optimistic that DOO-RE will play a significant role in advancing human activity recognition technologies, enhancing smart automation systems, and enabling the rapid setup of smart spaces through ambient sensors.

## Background & Summary

The proliferation of the Internet of Things (IoT) has made it possible for surrounding environments to provide intelligent services, including public safety^1^, environmental monitoring^2^, or independent living^3^. This necessitates the development of smart systems capable of interpreting and recognizing the intentions behind human activities. Such intelligent capabilities are derived from the analysis of data obtained from a variety of sources, including cameras^4^, wearable devices^5^, and ambient sensors^6^ within the realm of IoT. Recent research^7,8^ efforts have sought to integrate these data sources with machine and deep learning techniques to build intelligent systems in a way that demands less human manual effort. The studies highlight the growing need for data that is both privacy-sensitive and of high quality to enhance the effectiveness of these methods^9,10^. It is possible to inadvertently collect personal data that identify users during this process, despite their intentions, leading to privacy concerns. For example, video feeds inadvertently reveal personal characteristics like height or body shape^11^, even after blurring and the wearable devices capture sensitive information such as heart rates or app usage habits^12^. Gathering data via ambient sensors in an environment can provide a more generalized approach to data collection that respects privacy concerns, making it a preferable method for collecting data in real-world applications.

Table 1 describes datasets focused on ambient sensors, detailing the varieties of sensors used, the targeted activities, and the types of users without disclosing individual identities. These datasets primarily focus on personal environments, such as smart homes, and are tailored towards a narrow set of users. Their main objective is to recognize simple activities performed by solitary individuals. In contrast, public spaces^13,14^ offer a more complex scenario with a wider range of users and activities, including group interactions, posing greater challenges than those encountered in environments designed for individual use. This complexity in public settings has spurred considerable research interest^15,16^.

**Table 1:** Comparison of DOO-RE and related ambient sensor-based datasets.

Dataset name (years)	Ambient sensors	Target activities	Types of users
MERL (2007)^27^	over 200 motion sensors	Single Person Moving, Small Group Moving,	Single user,
		Small Meeting, Large Group Moving, Large Meeting	Group user
Wireless sensors Dataset (2008)^38^	14 wireless network nodes	Leave house, Toileting, Showering,	Single user
		Sleeping, Preparing breakfast, etc.	
CASAS (2009)^28^	Motion, Item sensor, Temperature,	Read a magazine, Hang up clothes,	Two separate
	Cabinet sensor, Burner sensor	Play a game, Set dining room table, etc.	users
OPPORTUNITY (2010)^39^	Switch, 3D acceleration sensor,	Groom, Relax, Prepare coffee,	Single user
	12 objects with 3D acceleration	Drink coffee, Prepare sandwich, etc.	
ARAS (2013)^40^	Force sensor, Distance, Photocell,	Going Out, Preparing Breakfast,	Two separate
	IR, Contact sensor, Temperature	Having Breakfast, Preparing Lunch, etc.	users
ContextAct@A4H (2017)^41^	Door, Light, Temperature,	Take Shower, Toilet use, Sleep, Cook,	Single user
	Co2 levels, Appliances states	Leave Home, Wash Dishes, Eat, Work	
E-care@home (2017)^42^	Motion, Light, Pressure,	Sitting, Moving, Watching TV, Burning,	Single user
	Luminosity, Heartbeat simulator	Exercising, Cooking, Eating, etc.	
Motionless	Accelerometer, Magnetometer,	Sleeping, Driving, Watching TV	Single user
Dataset (2022)^43^	Gyroscope, GPS, Microphone		
DOO-RE (2024)	Brightness, Humidity, Temperature,	Eating, Reading, Phone call, Seminar,	Single user,
	Sound, Podium, Door, Motion, Seat,	Lab meeting, Small talk, Studying together,	Group user
	Aircon, Light, Projector	Technical discussion, Eating together	

The existing datasets focus on personal spaces, such as smart homes, which consist of a small number of users and where only simple activities occur. Details of each dataset can be found in the corresponding citing paper.

With the growing fascination with public spaces, smart offices have emerged as a significant area of study, aimed at enhancing efficiency and effectiveness in user’s productivity^17,18^. For example, when preparing for an important presentation, the presenter can concentrate solely on the content, relying on intelligent services to manage ancillary tasks like activating the projector or dimming the lights. To the best of our knowledge, the current datasets^19–21^ related to office knowledge are limited to monitoring energy usage across workspaces, without addressing the recognition of user intentions or the development of user-centered office knowledge solutions. Addressing this gap, we have gathered data from ambient sensors in a meeting room, showcasing a smart office environment where activities, both individual and collaborative, are observed.

We introduce DOO-RE, a comprehensive dataset collected from a real meeting room, capturing continuous data streams 24/7 over 4 months from more than 20 students and faculty members. This dataset is distinct from those of smart homes due to the unique attributes of meeting rooms, such as their physical layout, which accommodates diverse activities in a single space, the typically larger number of users, and generally longer activity durations. Recognizing activities based on location alone (like previous studies) is challenging in such environments. To address this, we have equipped diverse types of sensors and categorized them into three types–device usage, user states, and environmental changes. DOO-RE’s data records include timestamps, device usage durations, and user count variations. Each user activity sample is compiled into an activity episode, with reliability ensured through detailed annotations focused on the activity’s purpose (e.g., Technical discussion), following a consensus among annotators and cross-verification. DOO-RE comprehensively captures various activities in a unique indoor meeting room environment, enabling sophisticated structured activity recognition and understanding of user intentions, essential for developing methods for smart office automation^17,18^ and other smart personalized services^22^. Furthermore, this dataset can contribute to optimizing sensor placement and data collection strategies necessary for establishing smart systems in other public spaces.

## Methods

### Dataset design

The rising importance of human activity recognition (HAR) led to the publication of over 80 HAR datasets in 2017 alone^23^. However, most of these focus on simple actions, such as sitting, using wearable technology^24–26^, which limits their applicability for broad IoT automation services. Table 1 highlights datasets with activity purpose-oriented labels suitable for IoT environments. The datasets mainly utilize ambient sensors for single or dual-user activities in private settings like smart homes. Although some datasets^27–29^ have explored multi-user scenarios, these are predominantly location-centric and do not fully address the nuanced needs of optimizing services in public spaces. We introduce DOO-RE, a dataset designed for a meeting room, employing ambient sensors to capture the distinct dynamics of the public space with two objectives as follows:

1. Establish a foundational understanding of collective user intentions in the presence of multiple users in a public space.
2. Explore diverse types of ambient sensor sequence patterns tagged with labels focused on the purpose of activities that help construct feasible activity recognition methods and optimized smart services.

Our dataset is titled *DOO-RE*, inspired by the traditional Korean term for *people cooperate*, reflecting the essence of a meeting room as a space for facilitating various group activities. A meeting room presents four *design considerations* compared to private spaces like homes: (1) Smart homes and meeting rooms differ significantly in their structural organization. Smart homes are segmented into distinct areas, each with a specific function, such as kitchens and bedrooms, allowing for easy activity monitoring through location-based sensors. Conversely, meeting rooms lack this physical segmentation, making it challenging to identify activities through location sensors or any single type of sensor alone. As a result, to understand the activities within a meeting room, it’s essential to incorporate data from various sensors, including those triggered by device usage (e.g., turning lights or projectors on and off) and environmental changes (e.g., variations in sound and temperature). This approach enables the differentiation of activities. (2) The composition of participants in a meeting can vary mid-session, introducing significant variation in participant behavior even within one activity. To address this without invading privacy, a combination of user-driven sensors, such as location and seat occupancy, is used to infer changes in participation. (3) Meeting rooms often witness multiple participants engaging in different activities simultaneously. To accurately capture these concurrent actions, it is crucial to record not just the commencement but also the duration of sensor activations, marking both their start and end times. This method allows for the identification of overlapping activities, for example, by tracking when two seats are occupied concurrently. (4) Meetings tend to last longer than household activities due to the higher number of attendees, necessitating an efficient data storage solution that records sensor state changes rather than continuous raw data. This selective recording strategy is vital to prevent storage overruns.

To accommodate the unique requirements of meeting environments and potential uses with other datasets, our dataset is designed to include diverse types of sensors, encompassing those driven by device operations, user actions, and environmental conditions. These three categories are common across almost all spaces and facilitate consistent integration or transfer with various data sources. For example, device operation data represents interactions with technological infrastructure, user action data reflects people’s behavior patterns, and environmental condition data records the physical state of the space. These categories support the development of integrated data analysis methodologies that are applicable across different research domains, enabling researchers to effectively use data in a broader and more varied set of environments.

DOO-RE carries out six label selection and validation steps for reliable activity labeling on data collected in the wild. This process is notably distinct from the annotation strategies not specifically detailed in existing datasets based on ambient sensors. Essentially, relying solely on a single expert for naming or annotating activities is deemed precarious in such settings due to the potential variability in names and perspectives for the same activity among different individuals. The approach involves ensuring consensus among a majority regarding the activity’s name and perspective, thereby aligning the annotation outcomes appropriately. This is achieved by engaging multiple annotators who, through collaborative discussion, synchronize terminology for each activity label. Subsequently, the annotators label unnamed episodes according to the agreed-upon activity labels and collectively validate the results of this labeling.

### Data collection setup

#### A meeting room condition

**Fig. 1:**
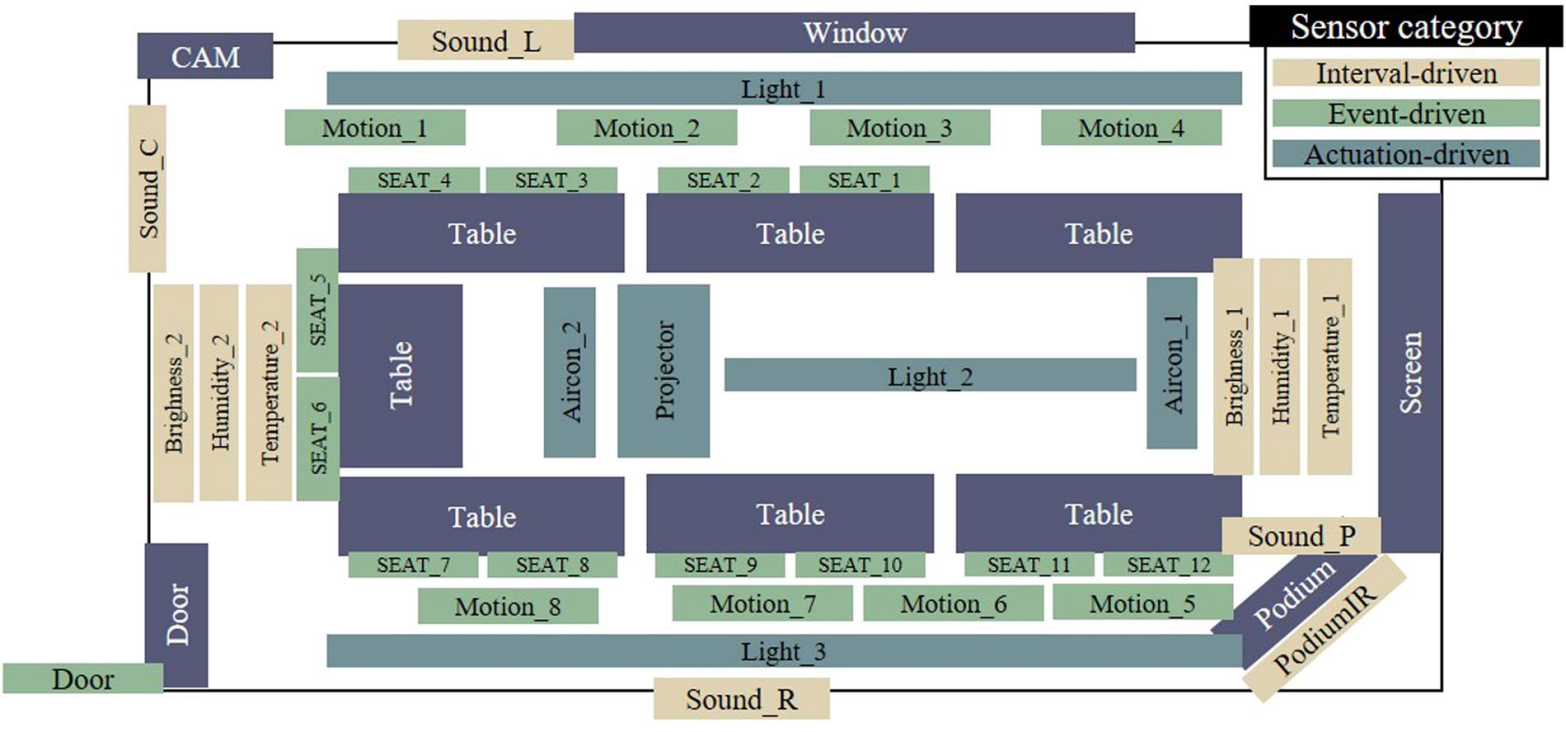
The meeting room testbed layout and installed location of ambient sensors. Essential meeting-related items, such as tables and a projector, are basically arranged. After observing how users interact within the meeting room, we select and install appropriate sensor types for each specific area.

Ambient sensors that detect data reflecting the sequences of users’ activities are deployed in a university meeting room. The layout of this room, depicted in Fig. 1, features a standard meeting area configuration with a single entrance, and seven elongated tables, each accommodating two seats. Additionally, it is equipped with a projector, a screen, and a podium to facilitate presentations. The orientation within the room is defined as left or right, depending on the direction a user faces towards the screen, with the side of the room featuring a window designated as the left. For instance, *Sound_L* indicates a sound sensor placed on the left side of the room.

The meeting room is primarily utilized by graduate students and faculty members, without any specific criteria such as age, race, or gender for entry and participation. Before collecting data, we briefed individuals using the meeting room about the data collection process and also posted notices. Throughout the data collection phase, we prominently displayed notices indicating that data was being collected and that individuals could request the deletion of their data if they wanted.

Unlike existing datasets, we do not provide any action guidelines to ensure the capture of realistic user behavior patterns within the space. Users naturally engage in activities according to their roles. Participants included over twenty students ranging from their 20 s to 30 s and three faculty members in their 50 s to 60 s. Consequently, we are able to obtain a diverse range of single or group activities.

#### Installed ambient sensors description

We utilize a variety of ambient sensors to discern the types of activities being undertaken by users. Our observation of the meeting room environment over a month reveals nine distinct activities, as detailed in Table 4. This observation guided our decision on the optimal placement of ambient sensors to capture user activities, illustrated in Fig. 1. The sensors fall into three distinct categories: Actuator-driven, User-driven, and Environment-driven.

- Actuator-driven sensors are designed to detect changes in the status of their associated actuators.
- User-driven sensors activate upon alterations in user states.
- Environment-driven sensors monitor changes in the surrounding environment.

Actuator-driven sensors (i.e. Aircon, Light, Projector) are connected to their respective smart devices to check if a device is being used. **Aircon** sensors monitor the operation of the air conditioners positioned at the front and rear of the room, installed on the ceiling. **Light** sensor, positioned next to the entrance on the wall, is connected to the light switch. This sensor oversees the status of three groups of lights (Left, Center, Right) on the ceiling, with each group comprising three lights arranged longitudinally from the front to the back of the room, and communicates this information back to the **Light** sensor. **Projector** sensor is linked to the projector, which is mounted in the middle of the ceiling.

Four types of user-driven sensors exist in the meeting room: Presenter Detection, In/Out, Motion, and Seat. **Presenter Detection** sensor, affixed to the podium’s front, identifies a presenter’s existence. **In/Out** sensor is located on the wall next to the door entrance to track users’ entry and exit. **Motion** sensors, mounted on every walls, monitor the movement paths of users within the room. **Seat** sensors, installed on every seat, ascertain whether a seat is currently occupied.

Our setup includes four types of environment-driven sensors: Sound, Brightness, Humidity, and Temperature. **Sound** sensors are strategically placed on each wall to determine the direction of target sounds. Additionally, they are fixed onto specific objects to detect particular user actions, such as in front of the podium to detect a presenter. **Brightness,** **Humidity** and **Temperature** sensors are strategically positioned in each sub-area to reflect variations in the internal environment influenced by external changes, such as weather shifts visible through windows. For each sensor type, one is located at the room’s front and another at the back, to gauge the influence of external conditions. These sensors provide indirect insights into the external environment of the meeting room, such as the time of day or physical climate, rather than directly detecting activities. This data helps other sensors fine-tune their activity recognition by adjusting their measurements based on these environmental cues.

When installing several sensors of the same kind in a single area, it is important to assign unique names to each sensor for differentiation, as illustrated in the **Sensor Name** section of Table 2. This distinction is achieved by appending numbers or letters to their names, which are then used in the dataset record file. The name assigned to each sensor significantly reflects its physical location.

**Table 2:** Classification of the different types of ambient sensors placed in the meeting room: **Actuator-driven sensors** are sensors that display status when users operate actuators.

Sensor Categorization	Sensor Type	Sensor Name
Actuator-driven	AirCon	Aircon_1, Aircon_2
	Light	Light_1, Light_2, Light_3
	Projector	Projector
User-driven	Presenter Detection	PodiumIR
	In/Out	Door
	Motion	Motion_1, Motion_2, Motion_3, Motion_4, Motion_5, Motion_6, Motion_7, Motion_8
	Seat	Seat_1, Seat_2, Seat_3, Seat_4, Seat_5, Seat_6, Seat_7, Seat_8, Seat_9, Seat_10, Seat_11, Seat_12
Environment-driven	Sound	Sound_L, Sound_C, Sound_R, Sound_P
	Brightness	Brightness_1, Brightness_2
	Humidity	Humidity_1, Humidity_2
	Temperature	Temperature_1, Temperature_2

**User-driven sensors** are aimed at detecting changes in the conditions of the users. **Environment-driven sensors** are designed to monitor variations in the surrounding environment.

**Fig. 2:**
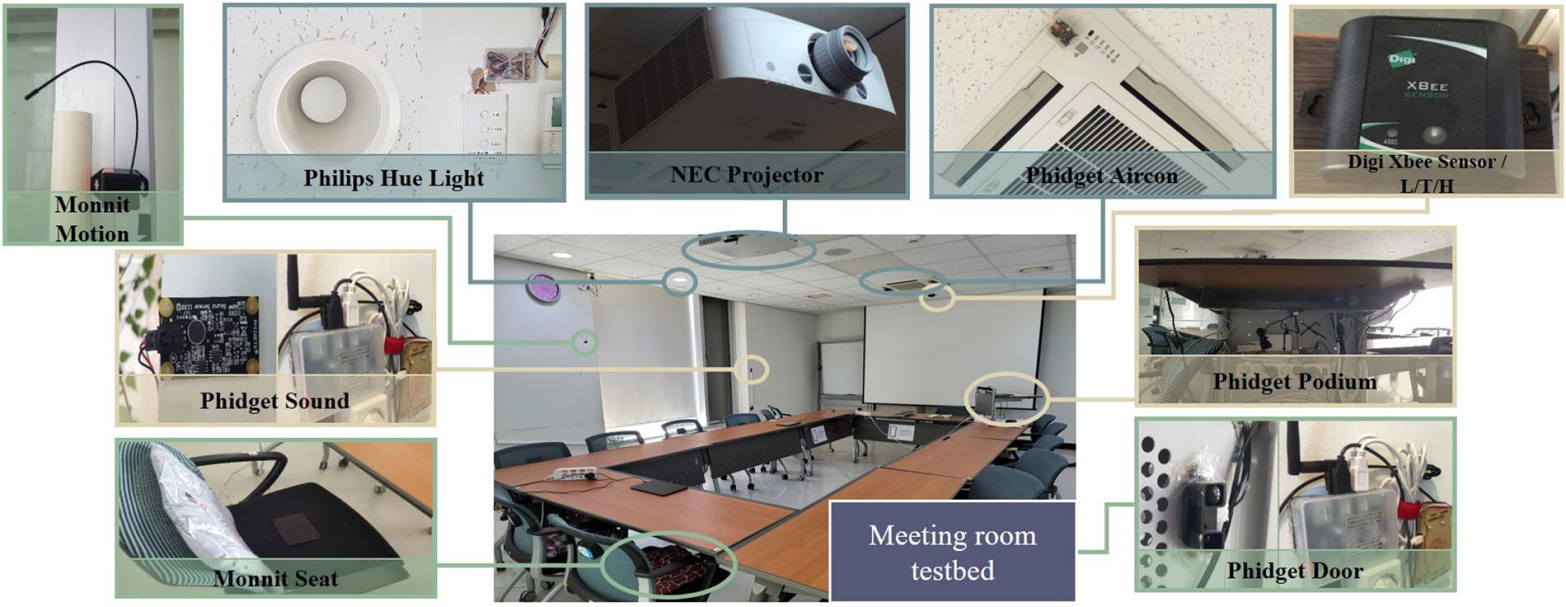
Image of sensors located in the meeting room testbed. *Phidget* and *API* are utilized for actuator-driven sensors. *Phidget* and *Monnit* are leveraged for user-driven sensors. *Digi XBee Sensor /L/T/H* and *Phidget* are used for environment-driven sensors.

Fig. 2 illustrates the sensor products utilized within the meeting room testbed to deploy the various sensor types previously discussed. A summary of product name, sensor type, publish time, and value range for each sensor type is described in Table 3. Our setup incorporates both wired and wireless sensors, with a preference for wireless sensors to ensure ease and stability in data collection. Wired sensors are employed exclusively in situations where appropriate wireless sensor alternatives do not exist or when issues such as battery depletion arise. Below are the specifics of the sensor products we use:

- **Light** sensor interfaces with the *Philips Hue API* to monitor the on/off status of three groups of lights (Left, Center, Right). **Projector** sensor keeps track of the projector’s power state via the *NEC Projector API*, which allows for detecting changes in projector usage.
- *Phidget*s are wired devices that are connected to Raspberry Pis for sensing infrared (IR) signals or sound. IR sensors are placed at the air conditioner, podium, and door locations to facilitate the sensing of **AirCon,** **Presenter Detection**, and **In/Out**, respectively. **Aircon** sensor, using Phidget IR controllers, reports the air conditioner’s power status based on operational changes. **Presenter Detection** sensor, relying on IR reflection, gauges distances from the podium every 10 seconds to track presenter movements. **In/Out** sensor, also using IR reflection, logs when individuals enter or leave a room, providing data useful for assessing the start and end of activities and estimating attendance. **Sound** sensors measure sound levels in decibels (dB) and update every 10 seconds to ensure reliable data collection.
- *Monnit* wireless sensors are deployed for detecting motion and seat occupancy. **Motion** sensors, with an 80-degree field of view, are mounted on walls to avoid interference, signaling movement through state changes. **Seat** sensors determine occupancy based on pressure applied to a plate, translating pressure into a binary seat occupied/unoccupied status.
- *Digi XBee Sensor /L/T/H* is a wireless integrated ambient light, temperature, and humidity sensor. It derives numerical values of **Brightness,** **Temperature**, and **Humidity** near the sensor. It transmits data to a Digi XBee Gateway via XBee network infrastructure. To conserve battery life, data transmission is set at 60-second intervals.

**Table 3:** Summary of product name, sensor type, publish time, and value range of each sensor. Publish times and value ranges vary according to the sensor types.

Product Name	Sensor Type	Publish Time	Value Range
Philips Hue (API)	Light	State Changes	On/Off
NEC Projector (API)	Projector	State Changes	On/Off
Phidget (Wired)	Aircon	State Changes	On/Off
	Presenter Detection	10 s	25–400
	In/Out	State Changes	Activate
	Sound	10 s	34–102 dB
Monnit (Wireless)	Motion	State Changes	True/False
	Seat	State Changes	True/False
Digi XBee Sensor /L/T/H (Wireless)	Brightness	60 s	0–1100
	Humidity	60 s	0–100%
	Temperature	60 s	15–30 °C

**Actuator-driven** and **User-driven (excluding Presenter Detection) sensors** have only two states as values and publish their states only when the values are flipped. On the other hand, **Environment-driven** and **Presenter Detection sensors** publish their states at regular intervals and their ranges of values are varied.

To manage and store sensor data efficiently, software agents on Raspberry Pis or mini PCs collect data points—a combination of sensor type, name, value, and timestamp—and send them to a database in a structured format (tuple). Before full-scale data collection, we confirm that the sensor measurement values align with the actual user’s behavior values, and record the timestamp from the sensors themselves to record the exact publishing time of the sensor values and send them to the database.

### Ethics statement

The Korea Advanced Institute of Science and Technology (KAIST) Institutional Review Board (IRB #KH2017-61) has granted approval for the acquisition of sensor data from the DOO-RE dataset. This approval process includes a thorough examination by the IRB of various aspects such as the data collection’s objectives, the participants involved, the methods used for collection, the environment where the data is collected, the types of data gathered, evaluations of safety, and mitigations for any side effects.

Prior to collecting data in a meeting room, participants are informed ahead of time. It is important to note that if participants do not give their consent, their data is removed from our database. We ensure that when capturing ambient sensor data, no information that could identify a user is stored. For the purpose of annotations, video data undergoes a blurring process to prevent user identification before it is stored in the database. We had no choice but to use video data to obtain the ground truth label accurately, but for data and privacy protection, the video data is made strictly viewable only by the annotators, and the video data is thoroughly removed after the annotation process is completed.

### Data annotation procedure

**Fig. 3:**
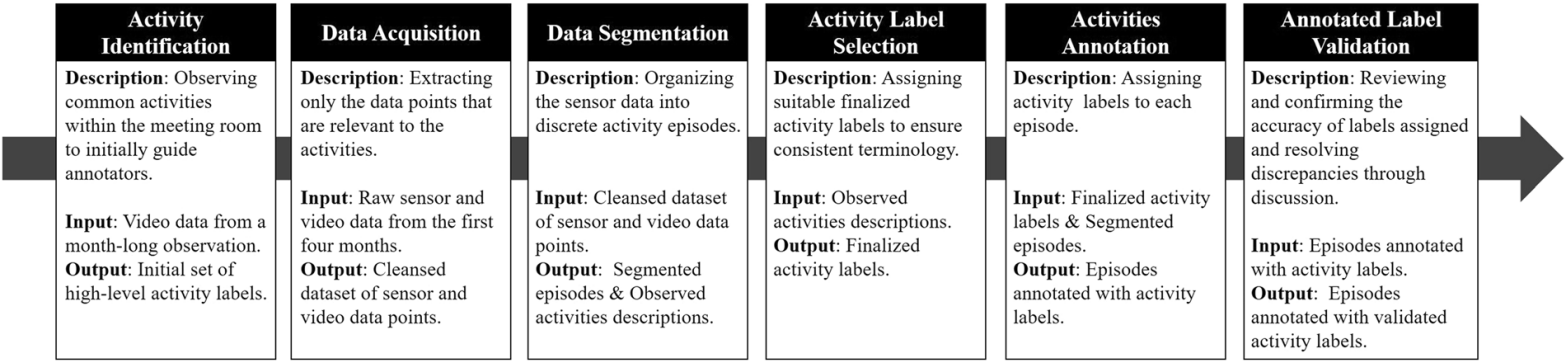
The overall activity annotation procedure of DOO-RE.

As shown in Fig. 3, the data annotation process involves three expert annotators in a meeting room environment, who follow a six-step methodology post data acquisition from installed ambient sensors: (1) Activity Identification, (2) Data Acquisition, (3) Data Segmentation, (4) Activity Label Selection, (5) Activities Annotation, and (6) Annotated Label Validation. This procedure helps to reproduce the classification of activities with minimal subjectivity, focusing on purpose-oriented activities in various types of spaces. Annotators leverage videos with ambiguous details to help differentiate activities that cannot be easily distinguished from sensor data alone.

#### Activity identification

To ensure an appropriate selection of activities and to lay down initial guidelines for annotators to follow, our approach begins with a month-long observation period within our testing environment to establish well-defined activity labels for smart automation systems. This step challenges us to determine the appropriate activity label level of detail. Our findings indicate that more granular labels often lead to the inclusion of irrelevant labels for our smart service goals, complicating the accurate assignment of labels by annotators and increasing subjective variability in labeling. Thus, we decide broader and purpose-oriented labels are better align with our research objectives, such as *Seminar* or *Small talk*. Even if a different type of meeting occurs in another public meeting room, specifying the type of meeting that appears in that space in this manner allows for the broad application of our annotation process across various data resources.

#### Data acquisition

To streamline the annotation process, we retrieve sensor data from our database, filtering out irrelevant data points for annotation. This involves keeping sensor data points structured as tuples {timestamp, sensor_type, sensor_name, sensor_value} and videos with obscured user identities. We acquire data and obtain meaningful data points for annotation through the following process: (1) We retrieve all sensor and video data from the first four months from our database. (2) If there are periods where video recording was not properly conducted, we discard the sensor data points from those periods. (3) If no one is present in the space, it implies that no activity has occurred, and such data is removed for the efficiency of annotation. We apply a deep learning-based human tracking method based on vision^30^ to identify periods when no one is present and discard both video and sensor data from these periods. (4) Ultimately, we are left with data points from periods when actual activities occurred in the meeting room, leaving us with only the meaningful data points for annotation.

#### Data segmentation

The purpose of the segmentation step is to organize the refined data sequences by defining the start and end times of each activity sample, termed as an episode. (1) A python-based software program roughly segments the data based on significant deviations in sensor readings from sensors such as *In/Out* or *Sound* sensors. For example, if the sound level is low and suddenly shifts to a high value, it can be seen as a point where an activity transition may have occurred. This timestamp can then be used as a reference to assume the start or end of an activity, allowing for a preliminary identification of activity start and end timestamps. (2) For more precise activity segmentation, human annotators begin to participate from this step onward. They watch the video recorded at those timestamps to determine the exact start and end times of each episode. As a result, each activity episode is accurately segmented, and the annotators record the start and end times of each episode in a spreadsheet. At this point, the episodes are still not labeled. The annotators also note down observed activities and their descriptions during this step for later reference in the label selection process. (3) This stage involves human intervention in data cleansing. Given the nature of the actual testbed, there may be missing or inaccurate sensor data, which the annotators directly verify. Detailed information about this can be found in the **Data quality** section. Additionally, after the annotators’ segmentation, episodes that are shorter than 5 minutes are removed, as 5 minutes is considered the minimum time for users to engage in meaningful activity in this setting.

#### Activity label selection

To maintain uniformity in the naming of activities, annotators review the activities identified during the segmentation phase and group similar activities under consistent terminology for the final activity labels. (1) Each annotator presents the activities they noted during segmentation. This step is crucial for covering all observed activities in the meeting room and ensuring a balanced perspective among annotators reviewing the same activities. (2) They categorize activities based on the similarity of the sequences of user behaviors and deliberate on suitable terms (i.e., labels) for each identified activity. Activities that are exceptional and infrequent within the meeting room are excluded. (3) Consequently, nine activity labels are established for annotation purposes: three designated for individual users and six for group activities, as detailed in Table 4. The activities are defined as follows:

- **Eating**: An individual enters and eats quietly at a table.
- **Phone call**: An individual moves around, stands or sits in the meeting room while making a phone call.
- **Reading**: An individual sits at a table to read or study.
- **Small talk**: Two or three individuals sit close to each other and engage in a casual conversation.
- **Studying together**: Two or more individuals collaborate in studying for an extended period.
- **Eating together**: Three or more individuals enter and dine together at several tables.
- **Lab meeting**: A regular progress meeting where, unlike in a seminar, multiple attendees present sequentially.
- **Seminar**: A session where a few invited speakers present extensively on a particular subject.
- **Technical discussion**: A group meeting aimed at addressing technical issues in projects without a designated speaker.

**Table 4:** The activity annotation resulting after the data annotation procedure.

Category	Activity name	# of episode	Average duration (sec)	Average # of participants
Single-user based	Eating	42	1440.60	1
	Phone call	150	807.15	1
	Reading	213	2439.25	1
Group-user based	Small talk	153	2786.79	3
	Studying together	21	4925.05	2
	Eating together	16	1482.44	3
	Lab meeting	29	4515.62	10
	Seminar	33	3332.82	5
	Technical discussion	39	3379.67	7

We find 9 activities in the meeting room: 3 for single-user and 6 for group-user based. A **single-user-based** activity is for one person to perform a single task objective, and a **group-user-based** activity is for several people to collaborate on one task. The number of episodes, average duration, and the average number of participants vary from activity to activity.

#### Activities annotations

During this step, annotators assign labels to segmented episodes. (1) The total episodes are divided equally among the annotators, making each one the primary annotator for their set of episodes. (2) The primary annotator watches the video segments and labels each episode by breaking down the video into beginning, middle, and end, to identify a general sequence of actions. Ensuring accurate annotations is a priority, allowing primary annotators sufficient time to accurately determine activity labels. (3) All episodes are eventually labeled, and the outcomes are documented, including any discussion points that emerge during annotation.

#### Annotated label validation

In this phase, the accuracy of labels given by primary annotators is assessed by secondary annotators who check episodes that were not previously labeled. (1) They review random video segments to verify the accuracy of the labels assigned by the primary annotators, aiming to decide within five minutes to ensure efficiency. (2) Any label disagreements are recorded. (3) When an episode consistently receives the same label from multiple annotators, this label is validated. (4) In cases of disagreement, a discussion takes place. If a consensus is reached, the episode is labeled accordingly; if not, it is excluded to preserve the quality of the dataset. (5) After all episodes are precisely labeled, the sensor data sequences for each episode are compiled, completing the DOO-RE dataset, which relies on sequences of ambient sensor data.

During the validation process among annotators, it is found that there is approximately 96% agreement on the activity labels assigned. Around 4% of the episodes demonstrate labeling inconsistencies, which are addressed through discussions among the annotators. These inconsistencies typically occur when the actions of users within a space or the tools they are using (such as mobile phones or laptops) are not clearly captured, or when actions occur in blind spots leading to unclear situations. In such cases, the final labels are determined either by majority votes among the annotators or, if consensuses cannot be reached, the episodes are discarded.

## Data Records

### Overall data record description

**Fig. 4:**
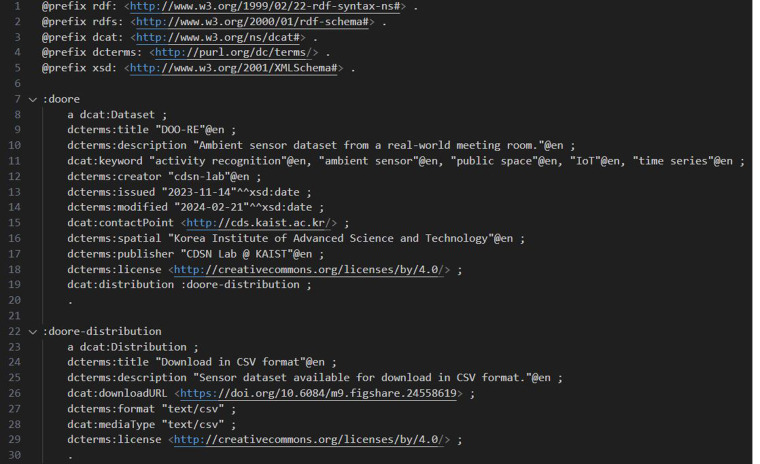
Data Catalog Vocabulary (DCAT) description for DOO-RE.

DOO-RE is accessible in the open access repository (10.6084/m9.figshare.24558619) along with its detailed description^31^. We provide DOO-RE’s overall metadata using Data Catalog Vocabulary (DCAT) (https://www.w3.org/TR/vocab-dcat-3/), as shown in Fig. 4, to enable integration with other private spaces’ datasets or potential uses with other data domain, thereby enhancing DOO-RE’s utility in evaluating activity recognition methodologies in both private and public settings. We share the overall information and distribution details of DOO-RE through the dataset and distribution properties of DCAT. According to the information provided in Table 4, the DOO-RE dataset encompasses nine distinct types of activities, each categorized by its number of episodes, average duration, and average participants. The dataset’s meeting room features a variety of sensors, categorized into actuator-driven, user-driven, and environment-driven types, with each activity utilizing a unique combination of sensors that align with its specific attributes. The dataset comprises 696 episodes totaling 452 hours (or 1,627,406 seconds) and involves 1,655 participants engaging in the activities, noting that individuals may be counted more than once. A summary of the characteristics of the collected dataset is summarized in Table 5.

**Table 5:** The summary of data collection results.

A summary of data collection	
Episode duration	Total: 1627406 sec (mean: 2338.22, median: 1443, stdev: 2379.93)
Number of episodes	Total: 696 (mean: 77.33, median: 39, stdev: 69.42)
Number of participants	Total: 1655 (mean: 2.38, median: 1, stdev: 2.73)
Activity categories	**Single-user based**: Eating, Phone call, Reading
	Group-user based: Small talk, Studying together, Eating together,
	Lab meeting, Seminar, Technical discussion
Categories of sensors	**Actuator-driven**: Aircon, Light, Projector
	**User-driven**: Presenter Detection, In/Out, Motion, Seat
	**Environment-driven**: Brightness, Humidity, Temperature, Sound

To describe the DOO-RE dataset’s features, we compute the total, average, median, and standard deviation for episode duration, number of episodes, and number of participants, respectively. Categories of activities and sensors appearing in DOO-RE are also organized for easy viewing at a glance.

**Fig. 5:**
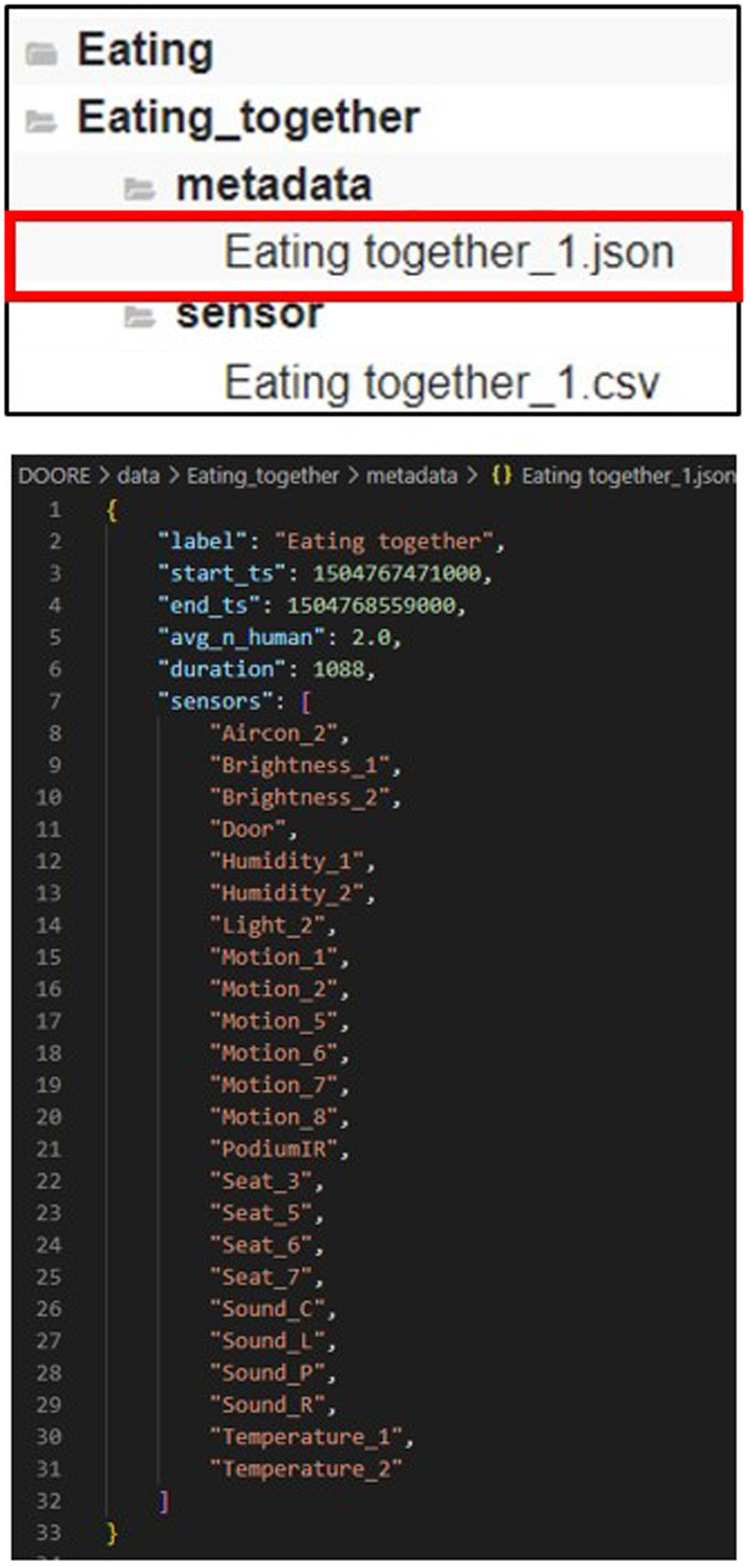
An example of data record structure and JSON-based metadata for each activity episode.

The organization of DOO-RE is hierarchical, with a root directory named *DOO-RE* that branches into nine activity-specific directories: *Eating*, *Phone call*, *Reading*, *Eating together*, *Lab meeting*, *Seminar*, *Small talk*, *Studying together* and *Technical discussion*. Within each activity directory, there are **metadata** and **sensor** subdirectories containing as shown in Fig. 5, respectively, metadata files and sensor data files for each activity episode. The naming convention for files within the *metadata* and *sensor* directories—formatted as ‘ < Activity_name > _ < index > .json’ and ‘ < Activity_name > _ < index > .csv’, respectively–establishes a direct correlation between the metadata files and sensor data files of each episode, based on their index. For example, the metadata file *‘DOO-RE/Eating_together/metadata/Eating together_3.json’* corresponds to the sensor data file *‘DOO-RE/Eating_together/sensor/Eating together_3.csv’* for the *‘Eating together_3’* episode, indicating that metadata files are in ‘json’ format and sensor data files in ‘csv’ format.

As described in Fig. 5, each episode’s metadata is provided in JSON (https://www.json.org/) files, offering concise details about the data contained in each activity episode’s CSV file. This JSON metadata is structured in a clear, key-value pair format, simplifying the identification and retrieval of data properties. The JSON format’s widespread compatibility with numerous programming languages and platforms makes it an ideal choice for data interchange and API communications. Metadata encompasses comprehensive details about each episode, including start and end timestamps, episode duration, average participant count, and the names of all sensors active during the episode. A sensor data file documents the actual sensor data values recorded during the episode.

The dataset operates on *Korea Standard Time* (KST, UTC + 9:00), reflecting the geographic location of the experimental meeting room in the Republic of Korea. In the other words, in order to obtain the human-readable date time at which a particular activity or sensor occurred, its timestamp must be converted to a human date based on the KST time zone. The timestamps are given in millisecond units to clearly distinguish between sensor data that is published almost simultaneously.

### Metadata

Metadata files provide supplementary information about each episode that sensor data files do not describe. These files allow for an overview of the episodes’ basic characteristics. A metadata file named ‘ < Activity_name > _ < index > .json’ includes the following information:

- **label**: This is the designated activity label for the episode.
- **start_ts** (msec): This represents the start time of the episode, with the timestamp adjusted for UTC + 9:00.
- **end_ts** (msec): This indicates the end time of the episode, also adjusted for UTC + 9:00.
- **avg_n_human**: This number reflects the average count of participants involved in each episode, measured during the data annotation procedure.
- **duration** (sec): This is the length of the episode, calculated by subtracting the start timestamp from the end timestamp and dividing by 1000.
- **sensors**: This is a compilation of the names of sensors that are active during the episode.

### Sensor data

A sensor data file has the data point sequences of one activity episode. Within this file, each data point is represented by three elements: **timestamp,** **sensor_name**, and **value** columns. The **timestamp** indicates when the sensor data is recorded. The **sensor name** is a term that can be identified between the sensors based on the location of each sensor, which is detailed in Table 2. The specific location tied to each **sensor name** is depicted in Fig. 1. The **value** represents the sensor’s condition at the recorded time, falling within the range specified in Table 3. As mentioned before, ambient sensors are categorized into three types: Actuator-driven, User-driven, and Environment-driven. To conserve storage space, data from Actuator-driven and User-driven sensors is stored only upon a change in their state, rather than recording every piece of raw data.

#### Actuator-driven

Actuators are devices that are manipulated by users within a designated area, with sensors dedicated to monitoring their operational status. DOO-RE incorporates data from three specific actuators: **AirCon,** **Light**, and **Projector**.

- **AirCon** - This involves sensors that monitor whether the air conditioning units are on or off. There are two sensors, **Aircon_1** and **Aircon_2**, responsible for tracking the operational status of air conditioners located at the front and back of the meeting room, respectively. An AirCon data point is registered whenever an air conditioner is turned on or off. The sensor data file lists all AirCon events under the *sensor_name* column as ‘Aircon_X’, where X is either 1 or 2. The status of each event, whether [On, Off], is noted in the *value* column of the CSV file.
- **Light** - This sensor identifies changes in the power state of the lighting and logs a data point whenever a change occurs. Lights are named according to their position within the space: **Light_1** for the light near the left wall, **Light_2** for the central light, and **Light_3** for the light near the right wall. A Light data event is recorded each time a light is switched on or off. The *sensor_name* column in the CSV files marks these events as ‘Light_X’, where X can be 1, 2, or 3, and their on or off status is detailed in the *value* column as [On, Off].
- **Projector** - The **Projector** sensor tracks the projector’s power status, creating data points each time the projector is powered on or off. These events are uniformly labeled as ‘Projector’ in the *sensor_name* column of the sensor data file, with their operational state [On, Off] specified in the *value* column.

#### Users-driven

User-driven sensors detect user state changes in the meeting room. The setup includes four distinct sensor categories: **Presenter Detection,** **In/Out,** **Motion**, and **Seat**.

**Fig. 6:**
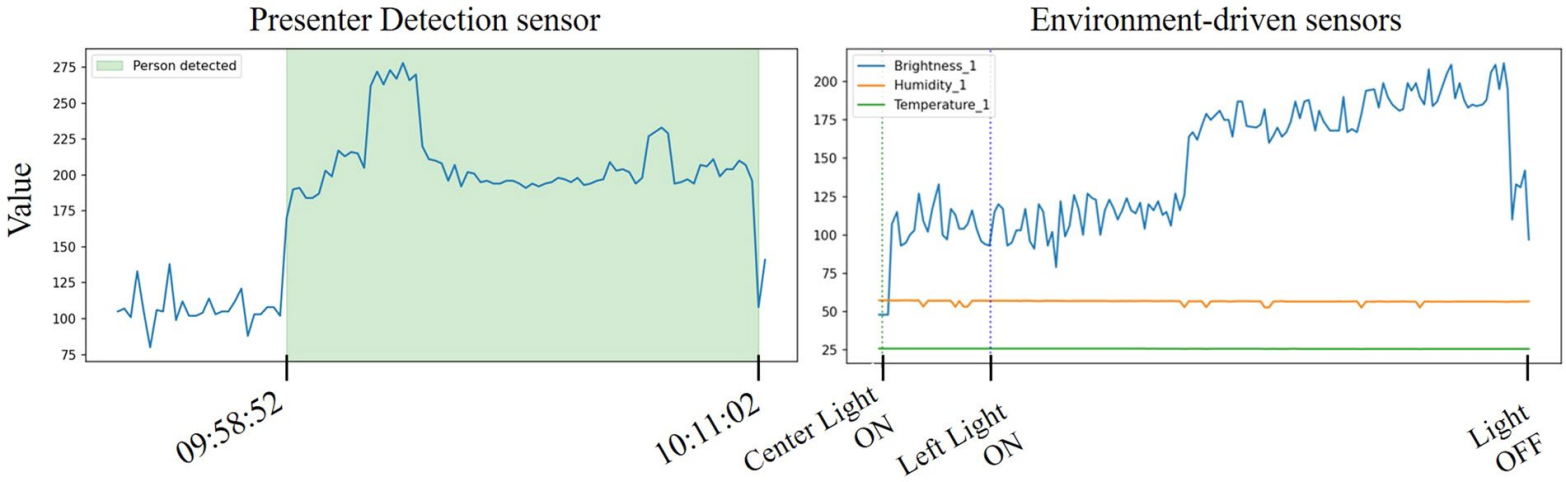
An illustration of a value change graph for the *Presenter Detection* sensor and environment-driven sensors (i.e. *Brightness*, *Temperature*, and *Humidity* sensors) in the file ‘Seminar_0.csv’. On the left, it is shown that the value of the **Presenter Detection** sensor escalates when a speaker is positioned in front of the podium. On the right, the illustration reveals the variations in states for each **environment-driven sensor** over a single episode.
- **In/Out** - This sensor is installed at the meeting room’s entrance to track user access and is denoted as **Door**. It signals an ‘active’ status upon detecting individuals nearby. Accordingly, these occurrences are recorded with ‘Door’ in the *sensor_name* column and ‘active’ in the *value* column of the CSV files.
- **Motion** - Distributed motion sensors, numbered **Motion_1** to **Motion_8**, identify movements in their vicinity. A ‘true’ status is reported upon movement detection, which switches to ‘false’ when no movement is observed. These data points are labeled as ‘Motion_X’ in the *sensor_name* column, with ‘X’ indicating the sensor’s specific location. Their statuses, whether [True, False], are documented in the *value* column of the CSV files.
- **Seat** - Sensors attached to each seat detect whether a seat is occupied by measuring the pressure exerted on the seat plate, returning ‘true’ when someone is seated and ‘false’ when the seat is vacated. These sensors are uniquely numbered from **Seat_1** to **Seat_12** and their data are presented as ‘Seat_X’ in the *sensor_name* column, with their occupancy status, whether [True, False], specified in the *value* column of the CSV files.

- **Presenter Detection** - Positioned at the front of the podium, this sensor gauges the proximity of objects to the podium and is capable of detecting distances up to 30 cm. The data collected by this sensor is labeled as **PodiumIR**. Typically, in the absence of a person near the podium, the sensor’s readings fluctuate between 50 and 150. Conversely, the presence of a person results in higher readings, as illustrated in the left figure of Fig. 6. Data from this sensor are cataloged under the label ‘Podium’ in the *sensor_name* column, with readings ranging from 25 to 400, noted in the *value* column of the CSV files.

#### Environment-driven

Sensors designed to monitor environmental conditions periodically record the state of the meeting room. This setup includes four types of sensors: **Sound,** **Brightness,** **Humidity**, and **Temperature**.

- **Sound** - The sound sensors in the room measure the level of sound pressure at various locations. Four sound sensors are placed within the meeting room, each designated by its specific location: **Sound_P** near the podium, **Sound_C** on the south wall, **Sound_R** on the right wall, and **Sound_L** on the left wall. These sensors detect louder sounds with higher values. The sensor data is cataloged in the CSV files under the *sensor_name* column as ‘Sound_X’, where ‘X’ denotes P, C, R, or L. The sound levels recorded range from 34 dB to 102 dB in the *value* column.
- **Brightness,** **Humidity,** **Temperature** - For these environmental factors, sensors are positioned at both the front and back of the meeting room. The names of the sensors on the front are suffixed with ‘1’, while those at the back are suffixed with ‘2’. Brightness sensors, **Brightness_1** and **Brightness_2**, assess the light intensity to gauge the brightness of their respective areas, with values spanning from 0 to 1100 where higher numbers indicate more light. Temperature sensors, **Temperature_1** and **Temperature_2**, measure the ambient temperature in degrees Celsius(C), with a range from 15 to 30 degrees indicating warmer temperatures with higher readings. Humidity sensors, **Humidity_1** and **Humidity_2**, evaluate the moisture in the air, with their values ranging from 0 to 100 percent relative humidity(%RH), where higher values signify more moisture. The data from these sensors are represented in the CSV files under *sensor_name* column as ‘Brightness_X’, ‘Temperature_X’, or ‘Humidity_X’, where ‘X’ is 1 or 2, and the corresponding values are logged in the *value* column. The right figure in Fig. 6 illustrates the variations in values of these sensors.

## Technical Validation

### Sensor distribution differences between activities

**Fig. 7:**
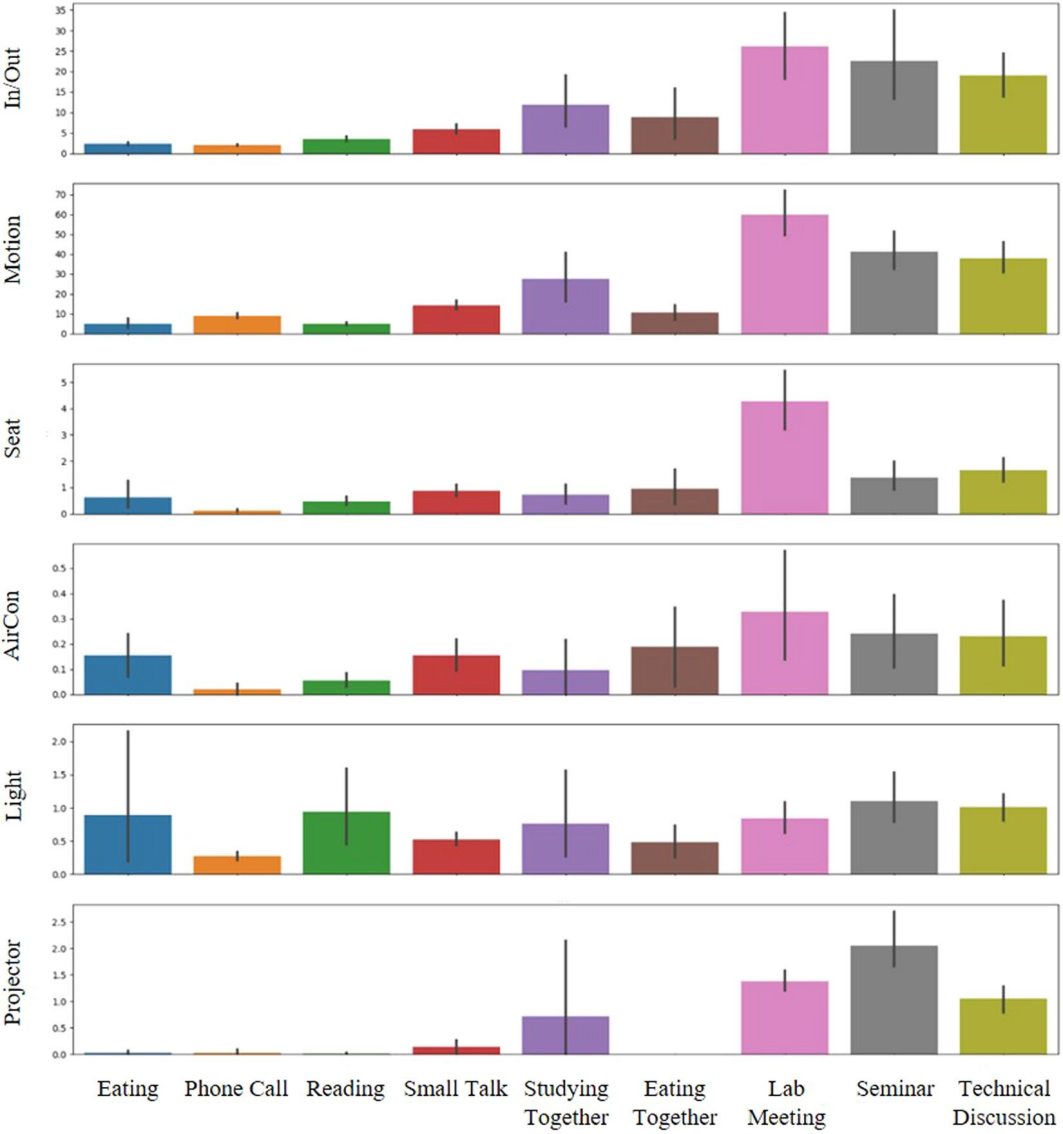
Frequency distributions box plots of *User-driven* and *Actuator-driven* sensors are presented per each episode. These sensors communicate their statuses using binary values, namely *True/False* or *On/Off*.

**Fig. 8:**
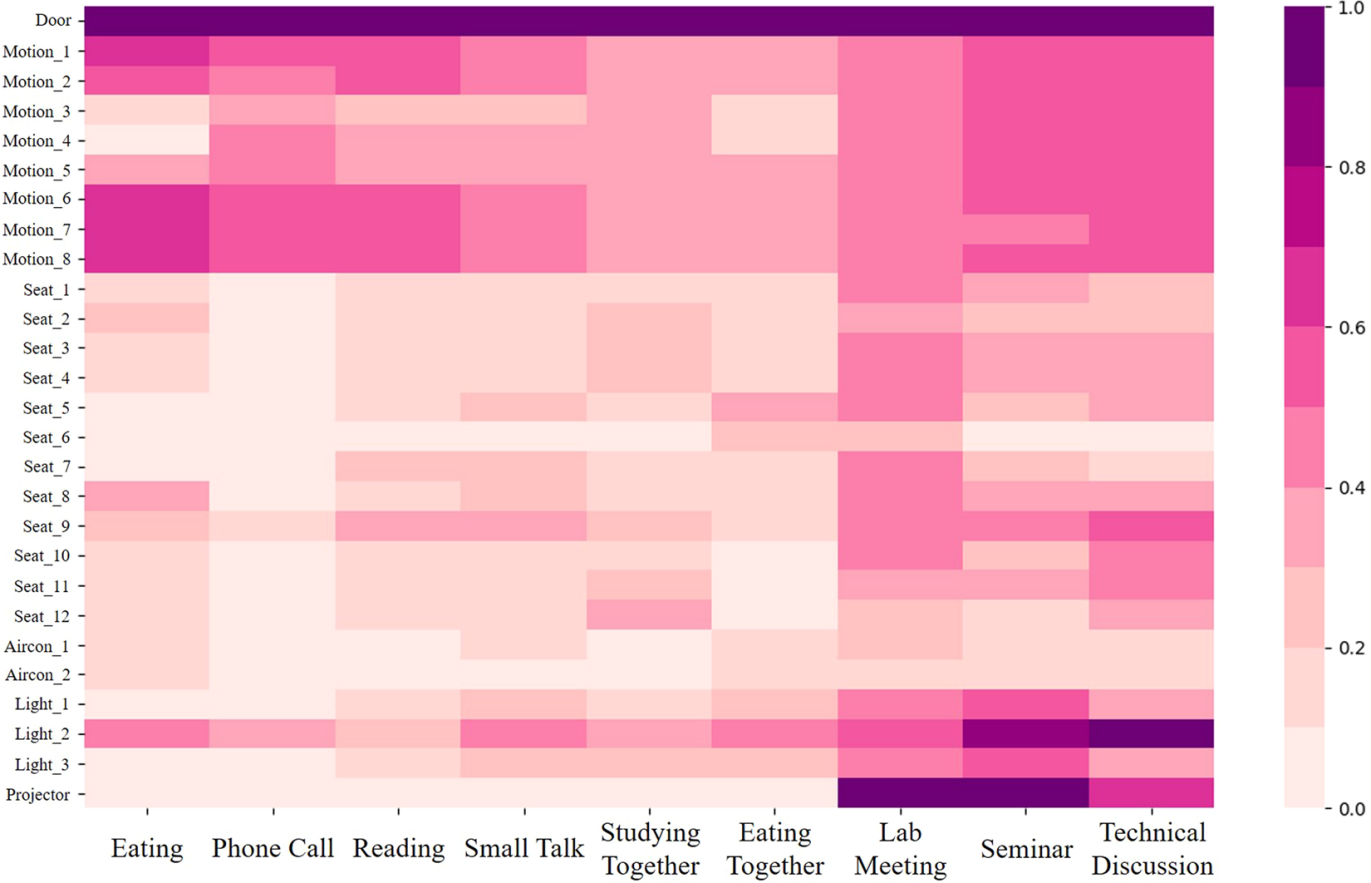
A heatmap visualization of whether each sensor occurs or not in an activity episode. The darker the color, the more likely a certain sensor appears in an activity episode.

**Fig. 9:**
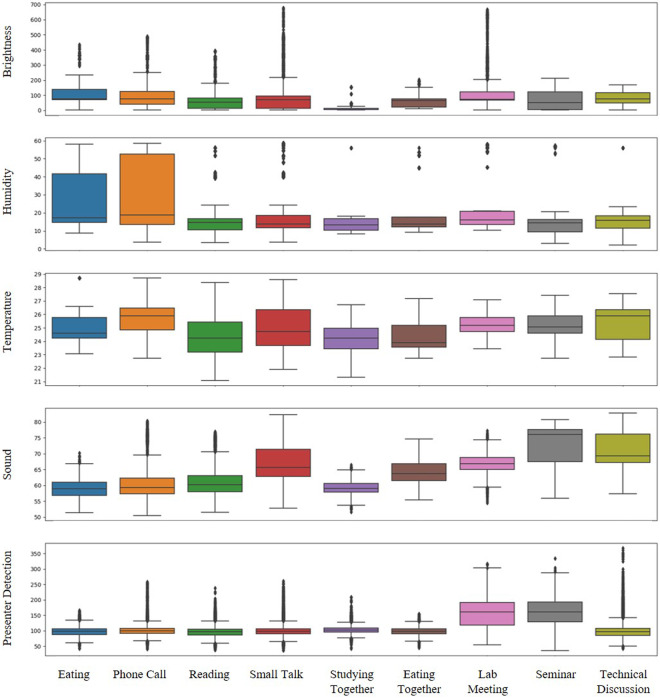
Value distributions of *Environmental-driven* and the *Presenter Detection* sensor. These sensors generate numerical state values, hence they are depicted through numerical distributions. Box plots are used to show the spread of values for each sensor across every episode of activity.

The average frequencies of *Actuator-driven* and *User-driven* sensors (excluding the *Presenter Detection* sensor) are depicted in Fig. 7. These sensors emit states in binary forms, such as *True* or *False*, which are more accurately represented through frequency counts (i.e., how often they occur). Fig. 8 shows in more detail whether each sensor name occurs per activity episode. Fig. 9 presents box plots that illustrate the value distributions for *Environmental-driven* sensors and the *Presenter Detection* sensor. Given that these sensors produce numerical state values, they are depicted using numerical distributions. Both figures reveal variations in sensor distributions and frequencies across different activities, suggesting that DOO-RE is highly effective in representing these activities. The observed patterns in sensor distributions and frequencies align with conventional wisdom.

Fig. 7 presents the frequency of occurrences for both *Actuator-driven* and *User-driven* sensors per episode. *User-driven Actuator-driven* sensors are activated once or twice at most, in contrast to *User-driven* sensors, which exhibit numerous activations within each episode. The activation rate of *Actuator-driven* sensors differs with the activity type and the specific actuators involved. The activation frequencies of the *AirCon* sensor are generally proportional to the number of participants in an activity. The *AirCon* sensor frequencies per episode are indicated under 1 since the data collection period of the DOO-RE dataset is from autumn to winter, when air conditioners are not used much. The *Light* sensor’s activation is linked to the length of an activity, becoming more prevalent in longer-lasting activities. Activities like *Phone Call*, which are brief and involve minimal interaction with devices, result in fewer activations of either the *Aircon* or *Light* sensors. Activities that utilize projectors, such as *Lab meeting*, *Seminar*, and *Technical discussion*, predominantly trigger the *Projector* sensor, aligning with expectations. The rate of activation for *User-driven* sensors, such as *In/Out*, *Motion*, and *Seat*, correlates with the participant count, indicating their utility in tracking user state changes during activities. They occur more frequently in large member-based activities. Moreover, the activation rate for these sensors is influenced by the activity’s duration. For example, activities like *Studying together* trigger more activations of the *In/Out* and *Motion* sensors compared to *Eating together*, due to the longer duration of the former. These differences help to distinguish between activities.

**Fig. 10:**
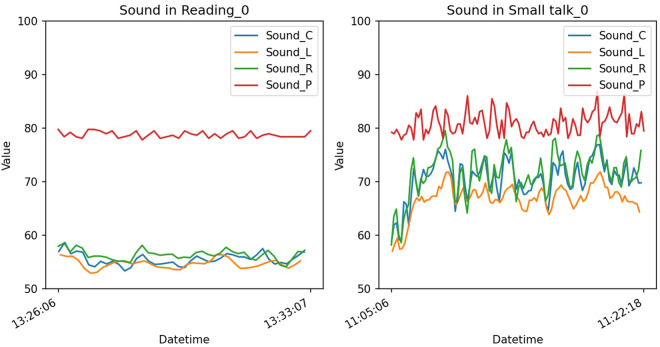
Changes of *Sound* sensors’ values in *Reading_0.csv* and *Small talk_0.csv* files. During the *Reading_0* activity, the *Sound* sensors record stable and low values, indicating a quiet environment. Conversely, in the *Small talk_0* activity, where room participants engage in conversation, the sensor data exhibits significant fluctuations, oscillating between high and low values.

Fig. 9 illustrates the variation in readings from the *Presenter Detection* and *Environmental-driven*’ sensors across different activities. As anticipated, the *Presenter Detection* sensor registers elevated readings in scenarios with a speaker, such as in a *Lab meeting* or a *Seminar*. Activities involving multiple users typically record higher *Sound* sensor readings, as these scenarios involve more individuals speaking. This is evidenced by the contrast in *Sound* sensor readings during *Reading* and *Small Talk*, as depicted in Fig. 10. During *Reading*, the *Sound* sensor consistently shows low readings, whereas in *Small Talk*, the readings vary widely as participants converse. The *Sound* readings remain low during the *Studying together* activity, reflecting its nature as a quieter group activity. Sensors for *Brightness*, *Humidity*, and *Temperature* exhibit comparable readings across various activities, attributed to their responsiveness to external environmental conditions like weather or time of day, rather than to the actions of the users. It is useful as additional information on how people behave in such external environments.

Cross-interpretation of Figs. 7, 9 together yields a more nuanced understanding of specific activities. Typically, activities involving groups tend to trigger higher readings from both *Actuator-driven* and *User-driven* sensors compared to activities with just one participant. Specifically, group activities such as *Lab meeting*, *Seminar*, and *Technical Discussion* show elevated sensor readings per episode across various sensors. This analysis reveals that by collectively examining data from diverse sensors, we can differentiate between activities that appear similar on the surface. For example, by analyzing data from *Presenter Detection* and *Projector* sensors, distinctions between *Seminar*, *Lab meeting*, and *Technical Discussion* become clear. In *Technical Discussion*, although *Projector* usage is similar to the other activities, *Presenter Detection* readings are significantly lower, reflecting the absence of a presenter. Similarly, *Reading* and *Studying together* may seem alike, but differences in *Sound* levels and *Motion* sensor activations allow for their differentiation. This demonstrates that diverse sensor applications enable the identification of distinct activities within a meeting space that cannot be separated into physically distinct areas.

### Statistical analysis between activities

We conduct a statistical analysis to understand the variation in sensor activation across various activities, as illustrated in Table 6. Considering the different sample sizes among activities, we perform an experiment using the t-confidence interval at a 95% confidence level to ascertain the precise differences between activities. This allows us to clearly verify the details mentioned above and provides ample clues for differentiation, as activities may share similar values across some sensors but display distinct ranges in at least one sensor. For example, the *Projector* is used in *Lab meeting* episodes, but not in every episode of *Eating together*. In addition, *Lab meeting*s and *Seminar*s seem broadly similar, but significant differences in values can be observed in *Motion* or *Seat* sensors. In activities involving a large number of participants, such as *Lab Meeting*, *Seminar*, and *Technical Discussion*, sensors are more likely to be activated compared to other activities.

**Table 6:** t-confidence interval at a 95% confidence level for each sensor of each activity.

Activity name	AirCon	Light	Projector	Presenter Detection	In/Out
Eating	[0.032, 0.253]	[−0.536, 3.298]	[0.000, 0.000]	[97.852, 98.68]	[0.958, 1.375]
Phone call	[0.001, 0.053]	[0.212, 0.414]	[0.000, 0.000]	[101.617, 102.329]	[0.946, 1.094]
Reading	[0.025, 0.088]	[0.064, 2.687]	[−0.005, 0.014]	[96.900, 97.172]	[1.507, 2.066]
Small talk	[0.080, 0.221]	[0.547, 0.982]	[0.003, 0.128]	[99.454, 99.786]	[2.513, 3.500]
Studying together	[−0.052, 0.147]	[0.019, 2.172]	[−0.167, 0.643]	[103.219, 103.77]	[2.674, 9.231]
Eating together	[0.057, 0.568]	[0.105, 1.520]	[0.000, 0.000]	[98.208, 99.381]	[0.966, 7.846]
Lab meeting	[0.135, 0.693]	[1.002, 2.308]	[0.779, 1.014]	[157.89, 159.487]	[8.821, 17.316]
Seminar	[0.064, 0.420]	[1.306, 2.876]	[0.743, 1.317]	[159.566, 161.176]	[5.764, 16.812]
Technical discussion	[0.074, 0.388]	[1.063, 2.014]	[0.375, 0.702]	[101.434, 102.476]	[6.829, 12.171]
**Activity name**		**Motion**	**Seat**	**Sound**	
Eating		[2.020, 31.361]	[−0.083, 3.178]	[63.507, 64.025]	
Phone call		[23.751, 51.102]	[0.218, 0.556]	[66.561, 66.956]	
Reading		[12.977, 22.328]	[1.161, 2.407]	[64.616, 64.809]	
Small talk		[38.332, 72.099]	[2.805, 5.862]	[69.276, 69.479]	
Studying together		[−28.348, 242.633]	[0.312, 8.354]	[70.166, 70.687]	
Eating together		[1.551, 85.199]	[−2.528, 12.778]	[68.731, 69.538]	
Lab meeting		[119.935, 372.823]	[12.199, 37.939]	[73.389, 73.775]	
Seminar		[63.070, 270.688]	[2.275, 14.513]	[70.584, 70.901]	
Technical discussion		[77.427, 232.778]	[4.879, 13.890]	[70.613, 70.954]	

This helps us understand the characteristics of each activity.

As indicated in Table 4, the number of episodes is disproportionate between activities due to the nature of the meeting room. Activities that use the meeting room relatively lightly, such as *Phone call*, *Reading*, and *Small talk*, occur more frequently than other activities. The average duration and the number of participants also vary depending on the type of activity. Activities based on single user typically have shorter durations compared to those involving groups. Within group-user-based activities, except for *Studying Together*, the average duration tends to increase with the average number of participants.

### Data quality

We investigate the data quality of ambient sensors in the DOO-RE for each activities. The data from *User-driven* and *Actuator-driven* sensors might not always be present in certain episodes, as their states are only published when users interact with the respective objects or devices. To distinguish between a sensor’s missing data and its inactive state, it becomes necessary to manually correlate video footage with sensor data to verify the sensor’s functionality. The findings on data quality are documented in the **data_quality.xls** file.

Generally, missing or inaccurate sensor data can arise from issues related to sensor batteries, network connections, physical damage to sensors, or malfunctions. For instance, the *Digi XBee sensor*, which measures **Brightness,** **Humidity**, and **Temperature**, is prone to rapid battery depletion, leading to data loss.

Beyond the issue of missing or incorrect data, sensors in public spaces often record data in unexpected ways due to various factors. Each sensor might exhibit missing or unexpected data for reasons including:

- Regulations at the university level might mandate the shutdown of air conditioners, irrespective of user preferences, preventing **Aircon** sensors from registering the *Off* state.
- Rapid, multiple operations of actuators (i.e. **AirCon,** **Light** and **Projector**) by users can prevent sensors from accurately tracking their status. For example, quickly turning a **Projector** on and off multiple times within a minute might result in the loss of the *Off* command due to the projector’s delay in turning on.
- The **In/Out** sensor, which uses IR distance to determine its state, might record unexpected values if a user approaches but does not enter a room, or if the user enters too quickly from a certain angle.
- The **Motion** sensor might miss detecting a person standing outside its detection angle or fail to capture rapid movement, resulting in missed or fluctuating values.
- The **Seat** sensor, which relies on pressure sensitivity, might register *True* values by merely moving a seat, regardless of actual occupancy. A certain **Seat** may not be correctly positioned in the location described in Fig. 1 because a user may change the position of the **Seat**, affecting the unexpected values of **Seat** sensors.
- **Sound** sensors, unable to differentiate between ambient noise and human voices, might record higher decibels due to non-voice noises like construction, leading to data that does not reflect the intended context.

We update the dataset’s repository by continuously updating the possible causes of the sensor value issue to help reproduce public space-based datasets like DOO-RE. Due to sensor data natural characteristics, they may contain erroneous data that cannot be visually verified, in which case additional data processing is recommended. Depending on the need, simple calibration methods such as data imputation, data smoothing, filtering, etc., can be employed.

## Usage Notes

### Potential applications

DOO-RE is instrumental in evaluating the resilience of human activity recognition applications in real-world scenarios, especially in the presence of groups. It lays the groundwork for identifying different behaviors in public settings by analyzing the occurrence, value, and duration of data from various sensors. The statistical insights presented in the Tables 4, 6 highlight key characteristics of different activities, providing valuable guidance for enhancing activity recognition models in public areas. Previous studies^32,33^ using DOO-RE show that schemes that work well on DOO-RE also suit well on existing ambient sensor-based datasets. The results show that DOO-RE has the potential to act as a helper to improve group activity recognition performance by providing multi-sensor perspectives of users’ behavior. Moreover, recent studies^34^ show DOO-RE’s utility in validating online recognition approaches.

Sensor-based recognition methods find applications in fields from healthcare to human-machine interaction^35^, and DOO-RE, by focusing on public spaces–a domain not extensively covered by existing datasets–plays a crucial role in broadening these applications. DOO-RE is also valuable for learning user preferences^22^ to tailor smart services more effectively. Although current research predominantly targets individual user scenarios, incorporating concepts of group dynamics^36^ into DOO-RE’s analysis of group behavior can pave the way for more adaptable and scalable smart services.

### FAIR principles

The FAIR Principles are a widely adopted set of guidelines related to data management^37^. FAIR stands for *Findable, Accessible, Interoperable, Reusable*, which are principles designed to facilitate the efficient use and management of research data. Each principle evaluates whether datasets are more useful, accessible, and easy to share. DOO-RE conforms to these principles for the following reasons:

- Findable - The data and metadata of DOO-RE are easily discoverable via a DOI (10.6084/m9.figshare.24558619)^31^, and metadata based on DCAT ensures rich and accurate searchability about DOO-RE.
- Accessible - DOO-RE’s data can be accessed without any restrictions through public repositories such as figshare and Zenodo. Metadata for the entire dataset, as well as detailed metadata for each sensor data, are always accessible in the repositories.
- Interoperable - DOO-RE uses DCAT format metadata to work along with other data. Data Catalog Vocabulary is an RDF vocabulary crafted to enhance interoperability among data catalogs that are available on the Web, making DOO-RE freely interoperable and exchangeable with other open data. Detailed metadata for each sensor data in JSON format is also provided, facilitating easy exchange with other datasets. Both DCAT and JSON are standardized languages, making it easy for other users to reuse.
- Reusable - Data should be clearly documented for reuse, and for this purpose, we publish this paper, *A dataset of ambient sensors in a meeting room for activity recognition*. This manuscript details Dataset design, Data Records, Technical Validation, Code Availability, etc., providing sufficient information about the data and enabling a variety of researchers to easily utilize it. The dataset and article are under a CC BY license (Creative Commons Attribution 4.0 International license) to ensure fair use, which is also stated in the open-access repositories.

### Supplementary information

DCAT data descriptor

data_quality worksheet

datas t-confidence interval worksheet

## Data Availability

The extraction of collected data, annotation of activities, conversion of collected data into formatted data record files, and analysis of sensor data utilize Python 3.7 and a variety of Python libraries. The codes are available on our lab’s GitHub site (https://github.com/cdsnlab/ScientificData). The DOO-RE dataset files and outcomes of data collection are hosted on the public repositories, accessible at figshare (10.6084/m9.figshare.24558619)^31^ and Zenodo (https://zenodo.org/records/7763477), with further inquiries welcome via contacting the corresponding authors. For optimal viewing, it is advised to open DOO-RE’s sensor data files, which are in CSV format, using Excel. JSON format metadata files can be easily viewed or modified using any text editor. Our MQTT-based IoT system (JAVA-based), named Lapras, gathers data from ambient sensors, actuators, and a camera. This system archives the raw data in a MongoDB database and uses Python to preprocess and extract sensor data for the creation of DOO-RE. Information and code for this collected system can also be requested through the lab’s website site or the corresponding author. We are open to assisting and providing information about DOO-RE, with the exception of requests for privacy-sensitive content, such as video recordings.
