# Author Correction: A dataset of ambient sensors in a meeting room for activity recognition

**DOI:** 10.1038/s41597-024-03478-8

**Published:** 2024-06-12

**Authors:** Hyunju Kim, Geon Kim, Taehoon Lee, Kisoo Kim, Dongman Lee

**Affiliations:** Korea Advanced Institute of Science and Technology, School of Computing, Daejeon, 34141 South Korea

**Keywords:** Research data, Scientific community, Social sciences, Research data, Scientific community, Social sciences

Correction to: *Scientific Data* 10.1038/s41597-024-03344-7, published online 21 May 2024

In this article the funding from ‘National Research Foundation of Korea (NRF) grant funded by the Korean government (MSIT) (No.NRF-2022M3J6A1063021, Industry and Society Demand Oriented Open Human Resource Development)’ was omitted. The original article has been corrected.

