# Supplementary material for: A dataset of ambient sensors in a meeting room for activity recognition
Source: Sci Data. 2024 May 21;11:516. doi: 10.1038/s41597-024-03344-7 (PMC11109118; doi:10.1038/s41597-024-03344-7)
Supplement: Supplementary file 1 — DCAT data descriptor [file 41597_2024_3344_MOESM1_ESM.pdf]

```
@prefix rdf: <http://www.w3.org/1999/02/22-rdf-syntax-ns#> .
@prefix rdfs: <http://www.w3.org/2000/01/rdf-schema#> .
@prefix dcat: <http://www.w3.org/ns/dcat#> .
@prefix dcterms: <http://purl.org/dc/terms/> .
@prefix xsd: <http://www.w3.org/2001/XMLSchema#> .

:doore
    a dcat:Dataset ;
    dcterms:title "DOO-RE"@en ;
    dcterms:description "Ambient sensor dataset from a real-world meeting room."@en ;
    dcat:keyword "activity recognition"@en, "ambient sensor"@en, "public space"@en,
"IoT"@en, "time series"@en ;
    dcterms:creator "cdsn-lab" ;
    dcterms:issued "2023-11-14"^^xsd:date ;
    dcterms:modified "2024-02-21"^^xsd:date ;
    dcat:contactPoint <http://cds.kaist.ac.kr/> ;
    dcterms:spatial "Korea Institute of Advanced Science and Technology"@en ;
    dcterms:publisher "CDSN Lab @ KAIST"@en ;
    dcterms:license <http://creativecommons.org/licenses/by/4.0/> ;
    dcat:distribution :doore-distribution ;
    .

:doore-distribution
    a dcat:Distribution ;
    dcterms:title "Download in CSV format" ;
    dcterms:description "Sensor dataset available for download in CSV format." ;
    dcat:downloadURL <https://doi.org/10.6084/m9.figshare.24558619> ;
    dcterms:format "text/csv" ;
    dcat:mediaType "text/csv" ;
    dcterms:license <http://creativecommons.org/licenses/by/4.0/> ;
    .
```
