# Supplementary material for: A dataset of ambient sensors in a meeting room for activity recognition
Source: Sci Data. 2024 May 21;11:516. doi: 10.1038/s41597-024-03344-7 (PMC11109118; doi:10.1038/s41597-024-03344-7)
Supplement: Supplementary file 2 — data_quality worksheet [file 41597_2024_3344_MOESM2_ESM.pdf]

	DoorAgent(acti	LightAgent	ProjectorAgent	AirconAgent	AmbientSensoı	AmbientSensorAgent(T,H)	MonnitServerAgent(Seat)	MonnitServerA	PodiumAgent (	PodiumAgent (	SoundAgent
Number of inco	87	0	5	5	0	0	0	0	0	0	0
Total number o	485	87	32	33	2500	10981	442	2878	3697	1262	21655
Number of epis	18	26	42	39	34	19	27	27	0	13	20

		DoorAgent(acti	LightAgent	ProjectorAgent	AirconAgent	AmbientSensor	AmbientSensor	MonnitServerA	MonnitServerA	PodiumAgent (	PodiumAgent (	SoundAgent
	Number of incorrect activa	0	0	0	0	0	0	0	0	0	0	0
	Total number of activation	3	4	0	2	208	460	10	49	70		2406
Eating	Number of episodes witho	1	0	2	1	1	0	0	0	0	1	0
	Number of incorrect activa	0	0	0	0	0	0	0	0	0	0	0
	Total number of activation	1	2	0	2	0	64	11	66	224	0	1798
Reading	Number of episodes witho	1	1	2	1	2	0	0	0	0	2	0
	Number of incorrect activa	0	0	0	0	0	0	0	0	0	0	0
	Total number of activation	0	2	0	0	0	28	1	63	94	0	750
Phone call	Number of episodes witho	2	1	2	2	2	0	1	1	0	2	0
	Number of incorrect activa	1	0	0	2	0	0	0	0	0	0	0
	Total number of activation	23	11	4	4	285	570	30	343	226	0	1811
Seminar	Number of episodes witho	0	0	1	1	1	0	0	0	0	2	0
	Number of incorrect activa	4	0	0	0	0	0	0	0	0	0	0
	Total number of activation	42	0	2	0	1276	2664	305	1932	813	0	6514
Lab meeting	Number of episodes witho	1	1	1	2	1	0	0	0	0	2	0
	Number of incorrect activa	0	0	0	0	0	0	0	0	0	0	0
	Total number of activation	3	3	0	4	0	40	14	102	135	0	1080
Technical discu	Number of episodes witho	0	0	1	0	1	0	0	0	0	1	0
	Number of incorrect activa	0	0	0	2	0	0	0	0	0	0	0
	Total number of activation	3	7	0	2	462	996	47	207	396	157	3167
Small talk	Number of episodes witho	0	0	2	2	1	0	0	0	0	1	0
	Number of incorrect activa	0	0		0							
	Total number of activation	8	6		2		5612			1343	932	2469
Study together	Number of episodes witho	1	1	2	1	2	1	2	2	0	1	1
	Number of incorrect activa	0	0	0	0	0	0	0	0	0	0	0
	Total number of activation	13	0	0	0	256	512	14	106	232	85	1659
Eating together	Number of episodes witho	0	2	2	2	1	1	1	1	0	1	0
	Number of incorrect activa	5	0	0	4	0	0	0	0	0	0	0
	Total number of activation	96	35	6	16	2487	10946	432	2868	3463	1244	21654
SUM	Number of episodes witho	6	6	15	12	12	2	4	4	0	13	1

		DoorAgent(acti	LightAgent	ProjectorAgent	AirconAgent	AmbientSensor	AmbientSensor	MonnitServerA	MonnitServerA	PodiumAgent (	PodiumAgent (	SoundAgent
Eating	Number of incorrect activa	2	0	0	0							
	Total number of activation	8	0	0	0							
	Number of episodes withc	1	2	2	2	2	2	2	2	0	0	0
Reading	Number of incorrect activa	4	0	0	0							
	Total number of activation	4	2	0	0							
	Number of episodes withc	2	1	2	2	2	2	1	1	0	0	0
Phone call	Number of incorrect activa	3	0	0	0							
	Total number of activation	5	3	0	0							
	Number of episodes withc	1	0	2	2	2	2	2	2	0	0	0
Seminar	Number of incorrect activa	30	0	3	0							
	Total number of activation	30	4	3	2							
	Number of episodes withc	2	1	2	1	2	2	2	2	0	0	0
Lab meeting	Number of incorrect activa	0	0	2	0							
	Total number of activation	51	12	3	2							
	Number of episodes withc	0	1	1	1	2	2	1	1	0	0	0
Technical discu	Number of incorrect activa	8	0	0	0							
	Total number of activation	55	6	2	1							
	Number of episodes withc	1	0	0	1	2	2	2	2	0	0	0
Small talk	Number of incorrect activa	6	0	0	0							
	Total number of activation	9	1	0	0							
	Number of episodes withc	1	1	2	2	2	2	1	1	0	0	0
Study together	Number of incorrect activa	0	0	0	0							
	Total number of activation	9	5	0	3							
	Number of episodes withc	0	0	2	1	1	0	2	2	0	0	2
Eating together	Number of incorrect activa	10	0	0	0							
	Total number of activation	10	3	0	1							
	Number of episodes withc	2	1	2	1	2	2	2	2	0	0	0
SUM	Number of incorrect activa	63	0	5	0	0	0	0	0	0	0	0
	Total number of activation	181	36	8	9	0	0	0	0	0	0	0
	Number of episodes withc	10	7	15	13	17	16	15	15	0	0	2

		DoorAgent(acti	LightAgent	ProjectorAgent	AirconAgent	AmbientSensor	AmbientSensor	MonnitServerA	MonnitServerA	PodiumAgent (	PodiumAgent (	SoundAgent
	Number of incorrect activa	0	0	0	0	0	0	0	0	0	0	0
	Total number of activation	4	4	0	0	2	4	2	2	2	2	0
Eating	Number of episodes witho	0	1	2	2	0	0	0	0	0	0	2
	Number of incorrect activa	2	0	0	0	0	0	0	0	0	0	0
	Total number of activation	10	0	0	0	1	4	1	1	2	2	0
Reading	Number of episodes witho	0	2	2	2	1	0	1	1	0	0	2
	Number of incorrect activa	0	0	0	0	0	0	0	0	0	0	0
	Total number of activation	1	2	0	0	1	4	1	1	2	2	0
Phone call	Number of episodes witho	1	1	2	2	1	0	1	1	0	0	2
	Number of incorrect activa	2	0	0	0	0	0	0	0	0	0	0
	Total number of activation	18	4	4	0	1	3	0	0	2	2	1
Seminar	Number of episodes witho	0	1	0	2	1	1	2	2	0	0	1
	Number of incorrect activa	8	0	0	0	0	0	0	0	0	0	0
	Total number of activation	96	4	6	2	1	4	0	0	2	2	0
Lab meeting	Number of episodes witho	0	1	0	1	1	0	2	2	0	0	2
	Number of incorrect activa	1	0	0	1	0	0	0	0	0	0	0
	Total number of activation	22	0	8	2	1	4	1	1	2	2	0
Technical discu	Number of episodes witho	0	2	0	1	1	0	1	1	0	0	2
	Number of incorrect activa	4	0	0	0	0	0	0	0	0	0	0
	Total number of activation	41	2	0	4	2	4	2	2	2	2	0
Small talk	Number of episodes witho	0	1	2	0	0	0	0	0	0	0	2
	Number of incorrect activa	1	0	0	0	0	0	0	0	0	0	0
	Total number of activation	3	0	0	0	2	4	2	2	2	2	0
Study together	Number of episodes witho	1	2	2	2	0	0	0	0	0	0	2
	Number of incorrect activa	1	0	0	0	0	0	0	0	0	0	0
	Total number of activation	13	0	0	0	2	4	1	1	2	2	0
Eating together	Number of episodes witho	0	2	2	2	0	0	1	1	0	0	2
	Number of incorrect activa	19	0	0	1	0	0	0	0	0	0	0
	Total number of activation	208	16	18	8	13	35	10	10	18	18	1
SUM	Number of episodes witho	2	13	12	14	5	1	8	8	0	0	17
